# Enhancing *Bacillus thuringiensis* Performance: Fertilizer-Driven Improvements in Biofilm Formation, UV Protection, and Pest Control Efficacy

**DOI:** 10.3390/microorganisms13030499

**Published:** 2025-02-24

**Authors:** Fan Zhao, Yufei Mao, Jiahong Yang, Sheng Yang, Xiong Guan, Zixuan Wang, Tianpei Huang

**Affiliations:** State Key Laboratory of Ecological Pest Control for Fujian and Taiwan Crops, Key Laboratory of Biopesticide and Chemical Biology of Ministry of Education, Biopesticide Research Center, College of Life Sciences, Fujian Agriculture and Forestry University, Fuzhou 350002, China; 18656905575@163.com (F.Z.); maoyufei0109@163.com (Y.M.); m13170891013@163.com (J.Y.); 18279872015@163.com (S.Y.); guanxfafu@126.com (X.G.)

**Keywords:** *Bacillus thuringiensis*, bacterial biofilm, fertilizers, wettable powders, biocontrol, insecticidal activity

## Abstract

This study investigated the effects of fertilizers on the biofilm formation, ultraviolet (UV) resistance, and insecticidal activity of *Bacillus thuringiensis* (Bt). *Bacillus thuringiensis*, a widely used microbial pesticide, has a minimal environmental impact and is highly effective against specific pests but is susceptible to environmental factors in field applications. Bacterial biofilms provide protection for Bt, enhancing its survival and functionality in the environment. However, the mechanisms by which fertilizers regulate the characteristics of microbial pesticides and enhance biofilm formation are not well understood. This study evaluated the effects of six fertilizers on the bacterial biofilm formation, the UV resistance, and the insecticidal activities of Bt wettable powders. The results demonstrated that fertilizers significantly enhanced the performance of three Bt preparations (Lv’an, Kang’xin, and Lu’kang). A compound fertilizer with 8.346 g/L of KCl, 2.751 g/L of ZnSO_4_·7H_2_O, and 25.681 μL/mL of humic acid was identified by response surface optimization, achieving the maximum BBF formation with OD_595_ value of 2.738. Furthermore, KH_2_PO_4_, HA, and ZnSO_4_·7H_2_O notably improved the survivability of Bt preparations under prolonged UV exposure, with the compound fertilizer providing the greatest protection. What’s more, fertilizers reduced the LC_50_ values of all Bt preparations, with the compound fertilizer decreasing the LC_50_ of the Lv’an Bt wettable powder to 0.139 g/L, a 3.12-fold increase in efficacy. This study demonstrated that fertilizers significantly enhance the UV resistance and insecticidal activity of Bt wettable powders by promoting bacterial biofilm formation. Herein, this study provides new strategies and theoretical support for Bt applications in modern sustainable agriculture.

## 1. Introduction

In the field of insect pest control, biopesticides have attracted much attention due to their numerous advantages, such as their minimal impact on the environment and specific effects on particular insect pests. Among them, *Bacillus thuringiensis* (Bt) has become one of the most widely studied, rapidly developed, and commonly applied microbial pesticides in recent years [1,2]. Bt is an insect pathogenic bacterium that utilizes toxin proteins to kill insect larval hosts. It has demonstrated remarkable efficacy in controlling Lepidopteran pests, especially in cruciferous crops, where *Plutella xylostella*, a major pest, causes significant economic losses due to its widespread resistance to chemical pesticides and ability to devastate yields [3,4,5,6,7]. However, Bt is affected by environmental stresses in practical applications, especially ultraviolet (UV) radiation, which greatly impacts its control efficiency [8,9]. Biofilms are critical structures that enable Bt to survive and function in the environment, providing protection against external stresses such as UV radiation and antibiotics while facilitating the dispersion and colonization of Bt cells [10,11,12,13]. Improving the stability and efficacy of Bt under field conditions remains an urgent challenge [14]. To address this challenge, researchers have developed various strategies to enhance the UV resistance of *Bacillus thuringiensis* biopesticides. These strategies include transcriptional responses, UV mutagenesis, biochemical pathways, and artificial approaches such as the addition of protectants and the development of recombinant *Bacillus thuringiensis* strains with stronger UV resistance [15,16,17,18]. These strategies operate through different mechanisms and aim to optimize the persistence and effectiveness of microbial pesticides in agricultural applications.

In response to the challenges encountered by Bt in application and based on previous research, this study aims to find better solutions. Research has found that Bacillus subtilis produces surfactants that trigger biofilm formation on the leaves of melons, thereby enhancing its biological control efficacy against plant pathogens. Thus, we identified bacterial biofilms as a potential natural protection. Biofilms consist of microbial cells and various endogenous extracellular polymeric substances (EPS). Their formation is a dynamic and reversible process, coordinated by interactions among different microbial species, and plays a crucial role in ecosystems [19]. As a natural protective barrier for bacteria, biofilms can shield them from external disturbances. This provides a novel and effective method to address the vulnerability of Bt to environmental factors during field applications [20,21]. The stability and function of biofilms play a key role in enhancing the persistence and effectiveness of Bt as a pesticide.

The formation of bacterial biofilms (BBFs) is of great significance to Bt. BBF acts as a protective barrier for bacteria, safeguarding them from external disturbances. This provides a novel and effective approach to addressing the vulnerability of Bt to environmental factors in field applications [22]. The stability and functionality of BBF play a crucial role in enhancing the persistence and effectiveness of Bt as a pesticide.

However, although previous studies have shown that biofilms play a critical role in enhancing the stability and activity of microbial pesticides, the mechanisms by which fertilizers regulate the characteristics of microbial pesticides and promote biofilm formation remain unclear. Biofilms provide protection for microorganisms, enhancing their survival in the environment and improving pest control efficacy [21]. In recent years, studies have found that the combined application of pesticides and fertilizers plays a significant role in preventing plant diseases and providing nutrients for crop growth. Compared to the long-term use of a single pesticide, combining pesticides with different mechanisms of action can produce synergistic effects in controlling plant diseases. A pesticide–fertilizer silica-based nanocomposite can synergistically control fungal diseases and provide nutrients to plants in an environmentally friendly way [23]. The combined use of pesticides and fertilizers has significant effects on maize seed pre-cultivation, soil cultivation, and pest control. However, the interaction between fertilizers and the biological processes related to Bt, particularly the formation of BBF and its subsequent impact on the biocontrol activity of Bt, has largely remained unexplored [24]. Understanding this complex relationship is of paramount importance as it has the potential to unlock new strategies for improving the performance and application of Bt in the agricultural environment.

To address this gap in knowledge, we propose a novel approach that combines fertilizer application with microbial biofilm technology, aiming to enhance Bt’s biofilm formation, UV resistance, and insecticidal activity. By integrating fertilizer application with microbial biofilm technology, we propose a novel approach to optimizing Bt’s performance in field conditions. This study systematically investigates the effects of six fertilizers, including KCl, KH_2_PO_4_, CAN, ZnSO_4_·7H_2_O, (NH_4_)_2_HPO_4_, and HA, on BBF formation, UV resistance, and the insecticidal efficacy of Bt preparations. Furthermore, using response surface methodology, we optimized a compound fertilizer that significantly enhances these key phenotypes.

## 2. Materials and Methods

*Fertilizers and Bt Preparations:* Six fertilizers were selected for this study: potassium chloride (KCl), potassium dihydrogen phosphate (KH_2_PO_4_), calcium ammonium nitrate (CAN), zinc sulfate heptahydrate (ZnSO_4_·7H_2_O), diammonium phosphate [(NH_4_)_2_HPO_4_], and humic acid (HA). Humic acid products were purchased from Shenzhen Dogo Biotech Co., Ltd. (1 kg liquid) (Shenzhen, China). These fertilizers were chosen based on their common use in agriculture, their potential to promote bacterial biofilm formation, and their minimal environmental impact. The chemical components of these fertilizers were considered for their ability to support bacterial growth, enhance biofilm stability, and increase the efficacy of *Bacillus thuringiensis* preparations. We also selected fertilizers that align with sustainable agricultural practices, ensuring that their use would not cause excessive environmental harm. Bt preparations used in this study were all WPs from Fujian Green Shell Bio-pesticide Co., Ltd. (Lv’an) (Nanping, China) with activity units of 32,000 IU/mg and 16,000 IU/mg, King Biotec Corp. (Kang’xin) (Wuhan, China) with an activity unit of 50,000 IU/mg, and Shandong Lukang Biological pesticide Co., Ltd. (Lu’kang) (Dezhou, China) with an activity unit of 32,000 IU/mg. The results of 32,000 IU/mg WP from Lv’an are presented in the main text, while the detailed results of other WP are provided in the Appendix A.

*Fertilizer Treatment and Biofilm Formation:* To investigate the effects of fertilizers on the BBF formation of Bt preparations, each Bt WP was used as a control (CK), and the addition of the above fertilizers to each Bt preparation in 12-well plates was used as the treatment groups. The 12-well plate was placed in an incubator and cultured under conditions of 52% humidity and 30 °C for 48 h. The BBF formation was quantified using a crystal violet staining method. Briefly, the supernatants of the cultured samples were discarded, and the pellets were washed with sterile distilled water to clean the planktonic bacteria and air-dried. Then, they were stained with crystal violet. After decolorization, the OD_595_ nm values were determined. The test included three replicates per sample and was repeated three times.

*Optimization of Fertilizer Concentration:* To optimize the concentrations of fertilizers that enhance biofilm formation, we used a multi-step approach combining the Plackett–Burman (PB) design, Steepest Ascent Method, Box–Behnken Design (BBD), and Response Surface Methodology (RSM). These methods allowed us to efficiently screen for the most effective fertilizers and fine-tune their concentrations for optimal biofilm formation [25].

(1) PB design

This initial screening method was employed to identify which fertilizers most significantly influenced biofilm formation. The six fertilizers (coded as A to F) were tested at two levels of concentration, as outlined in Table 1.

(2) Steepest Ascent Method

Based on the PB design, this method was used to refine the concentration ranges for the most influential fertilizers, guiding us to the optimal concentration range for further testing. 

(3) BBD

Based on the above results of the Steepest Ascent Method, a three-factor and three-level experimental scheme was designed (Table 2) with three repetitions of the central point to set up 15 experiments. Then, the RSM plots were generated.

(4) Response Surface Methodology (RSM)

RSM was employed as the final step to analyze and integrate the data from the PB design, steepest ascent method, and BBD. A quadratic regression model was established to evaluate the interaction effects among the key factors and to predict the optimal fertilizer combination for BBF formation. The model’s significance was validated using ANOVA, and the fitness of the model was assessed by comparing the predicted and experimental values.

*Modulation of UV resistance of Bt preparations by fertilizers*

Each fertilizer was added to 5 g/L of the Bt preparation. Then, the mixture was incubated at 52% humidity and 30 °C for 48 h. The Bt preparation without fertilizers was used as the CK. The samples were placed in the UV crosslinker to irradiate the UV wavelength of 254 nm for 1 h, 2 h, and 4 h, respectively. The viable bacterial rate was determined by the viable counting method.

*Effect of fertilizers on the insecticidal activity of Bt preparations*

The second instar larvae of the AD strain of *P. xylostella* were selected to determine the effect of fertilizers on the insecticidal activities of Bt preparations using the drug film method. Thirty insects were tested in each group [26].

*Statistical Analysis*

Data were analyzed using GraphPad Prism 9.0 for statistical significance. One-way and two-way ANOVA were performed, followed by Bonferroni correction for multiple comparisons.

Differences were considered statistically significant at *p* < 0.05. The data from the RSM were analyzed using Design Expert 13.0, and contour plots were generated to visualize the interactions between fertilizer factors.

SPSS 27.0 was used to calculate LC50 values and their confidence intervals. All treatments were performed in triplicate (*n* = 3), and the entire experiment was repeated three independent times to ensure reproducibility. Results are expressed as the mean ± standard deviation (SD), and statistical significance was set at *p* < 0.05.

## 3. Results

### 3.1. Fertilizers Significantly Promote Biofilm Formation of Bt Preparations

**Figure 1:**
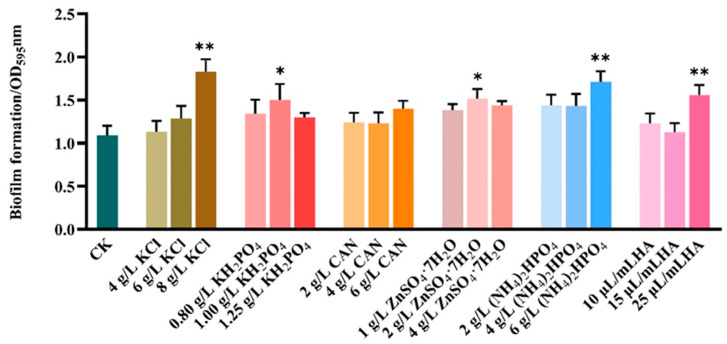
Effects of fertilizers on the BBF formation of 32,000 IU/mg Bt WP from Lv’an company. “*” indicated a significant difference (*p* < 0.05), while “**” indicated a highly significant difference (*p* < 0.01). All treatments were compared with the CK group.

**Figure A1:**
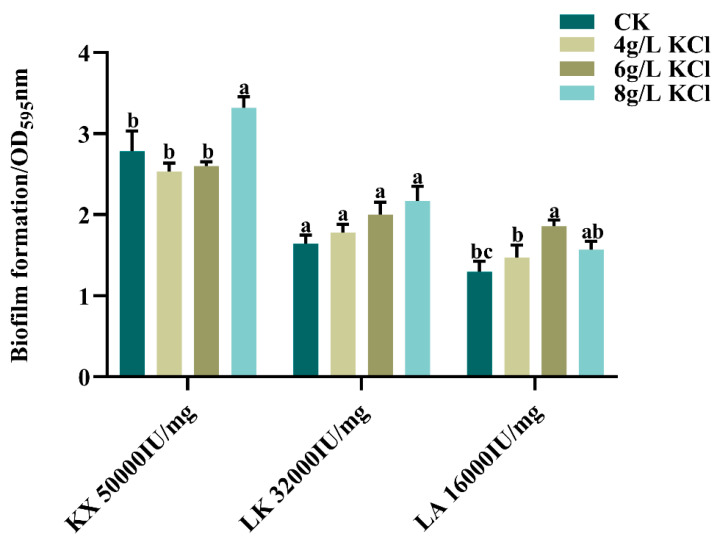
Effect of KCl on the biofilm formation of Bt WPs. KX: Kang’xin; LK: Lu’kang; LA: Lv’an. The following figures were the same. The presence of the same letter indicated no significant difference, and the presence of different letters indicated a significant difference (*p* < 0.05).

**Figure A2:**
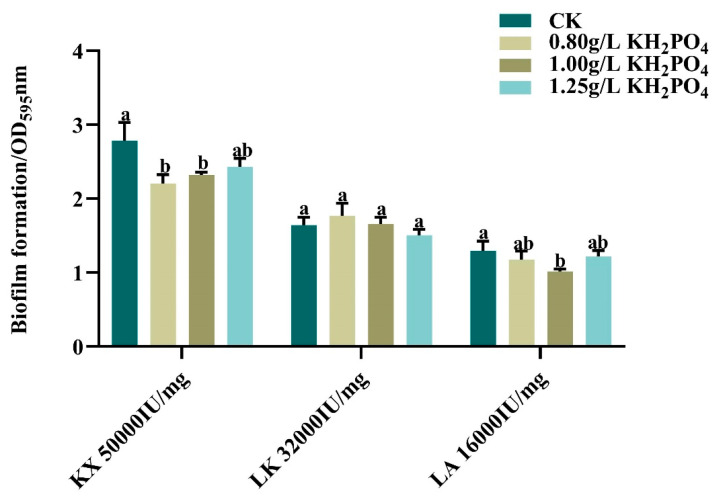
Effect of KH_2_PO_4_ on the biofilm formation of Bt WPs. The presence of the same letter indicated no significant difference, and the presence of different letters indicated a significant difference (*p* < 0.05).

**Figure A3:**
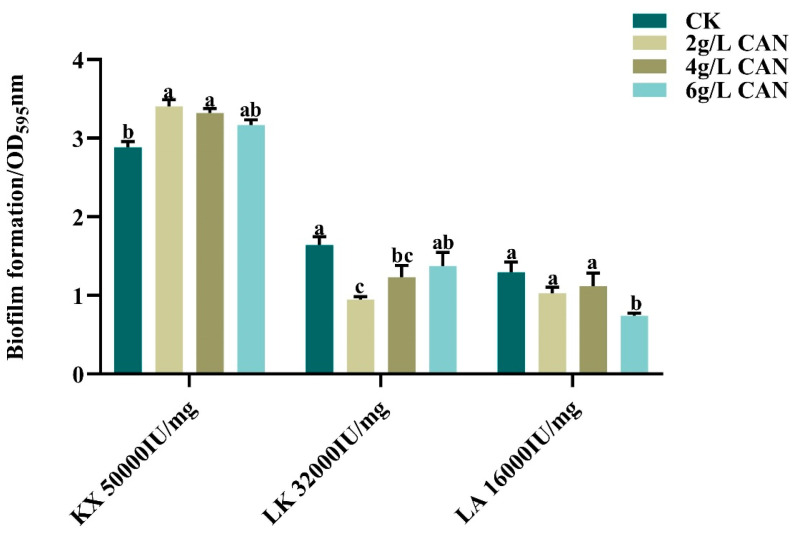
Effect of CAN on the biofilm formation of Bt WPs. The presence of the same letter indicated no significant difference, and the presence of different letters indicated a significant difference (*p* < 0.05).

**Figure A4:**
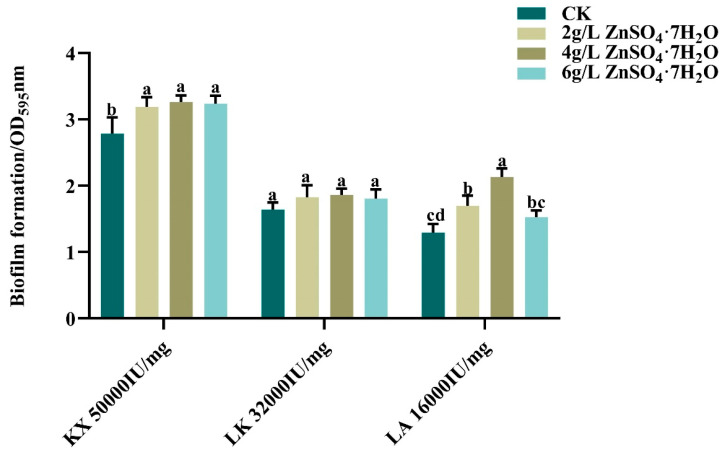
Effect of ZnSO_4_·7H_2_O on the biofilm formation of Bt WPs. The presence of the same letter indicated no significant difference, and the presence of different letters indicated a significant difference (*p* < 0.05).

**Figure A5:**
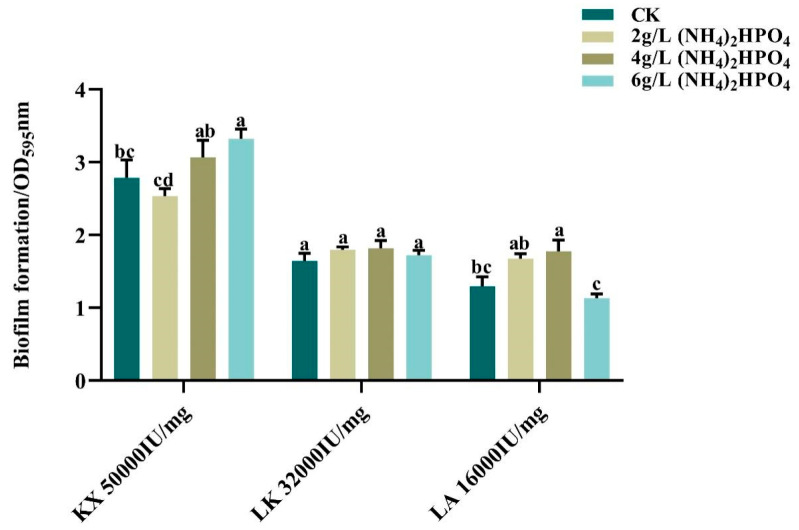
Effect of (NH_4_)_2_HPO_4_ on the biofilm formation of Bt WPs. The presence of the same letter indicated no significant difference, and the presence of different letters indicated a significant difference (*p* < 0.05).

**Figure A6:**
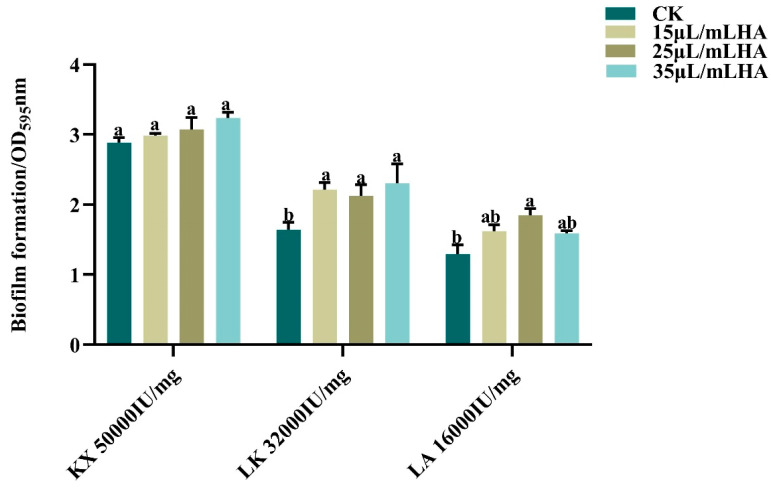
Effect of HA on the biofilm formation of Bt WPs. The presence of the same letter indicated no significant difference, and the presence of different letters indicated a significant difference (*p* < 0.05).

To investigate the role of fertilizers in regulating the ability of 32,000 IU/mg Bt WP of Lv’an company to form biofilms, different concentrations of fertilizers were mixed with the preparation. They were taken out for the quantitative determination of biofilm after 48 h of static incubation. As shown in Figure 1, 8 g/L of KCl or 25 μL/mL of HA highly significantly promoted the BBF formation of the preparation (*p* < 0.01), while 1 g/L of KH_2_PO_4_ or 6 g/L of (NH_4_)_2_HPO_4_ had a significant promotional effect. CAN had no effect on the BBF formation. Similar experiments were also conducted with other commercial Bt WPs (Figure A1, Figure A2, Figure A3, Figure A4, Figure A5 and Figure A6). Interestingly, the biofilm formation response to fertilizers varied among different commercial preparations. More specially, Kang’xin WP showed a strong response to KCl or ZnSO_4_·7H_2_O, while Lu’kang WP responded more strongly to KH_2_PO_4_ and HA (see Appendix A for details).

### 3.2. Fertilizers Improve UV Resistance of Bt Preparations

**Figure 2:**
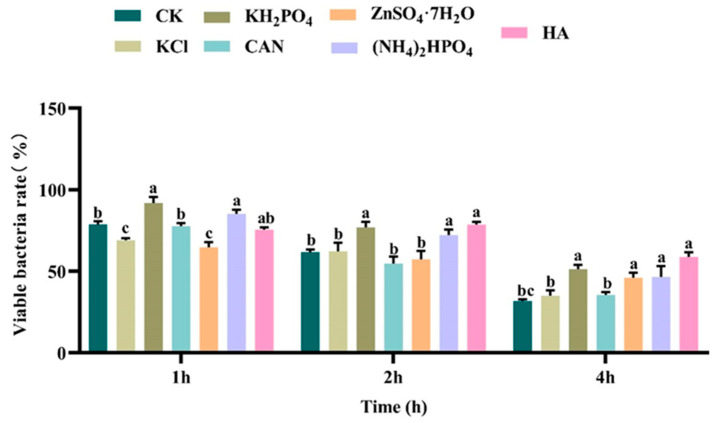
Effects of individual fertilizer on the UV resistance of 32,000 IU/mg Bt WP from Lv’an. The letters on the bar chart indicated the different significance of different samples. The presence of the same letter indicated no significant difference, and the presence of different letters indicated a significant difference (*p* < 0.05).

The impact of individual fertilizers on the UV resistance of 32,000 IU/mg Bt WP from Lv’an was investigated by incubating the Bt preparation with six different fertilizers. As illustrated in Figure 2, certain fertilizers significantly enhanced the UV resistance of the Bt preparations. Specifically, KH_2_PO_4_ (1 g/L) and (NH_4_)_2_HPO_4_ (6 g/L) showed a marked improvement in UV resistance after one hour of exposure, with survival rates significantly higher than those of the control group. This suggests that these fertilizers may help maintain the viability of Bt under UV stress, possibly by enhancing DNA repair mechanisms or stabilizing bacterial membranes.

After two hours of UV exposure, the protective effects of KH_2_PO_4_, (NH_4_)_2_HPO_4_, and HA (25 μL/mL) became even more pronounced, with these fertilizers exhibiting significant improvement in UV resistance. The continued effectiveness of these fertilizers under prolonged UV exposure indicates their potential role in mitigating UV-induced cellular damage, allowing Bt to better withstand environmental stresses.

After four hours of UV exposure, all the fertilizers tested, except for KCl and ZnSO_4_·7H_2_O, continued to enhance the UV resistance of Bt significantly. This finding indicates that KH_2_PO_4_, (NH_4_)_2_HPO_4_, and HA provide robust protection against prolonged UV exposure, maintaining the survival and efficacy of the Bt preparation.

**Figure A7:**
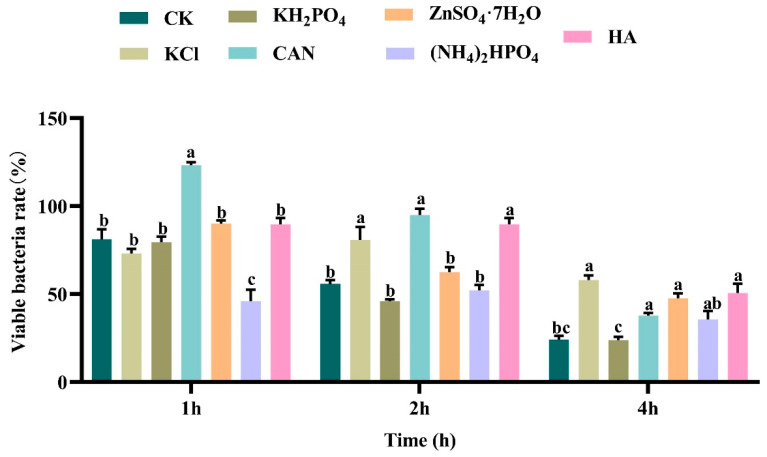
Effect of individual fertilizer on the UV resistance of 50,000 IU/mg Bt WP from Kang’xin. The presence of the same letter indicated no significant difference, and the presence of different letters indicated a significant difference (*p* < 0.05).

**Figure A8:**
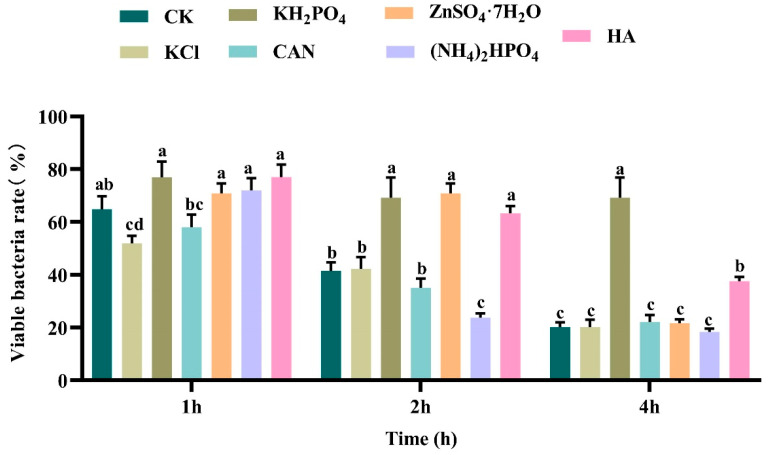
Effect of individual fertilizers on the UV resistance of 32,000 IU/mg Bt WP from Lu’ka. The presence of the same letter indicated no significant difference, and the presence of different letters indicated a significant difference (*p* < 0.05).

**Figure A9:**
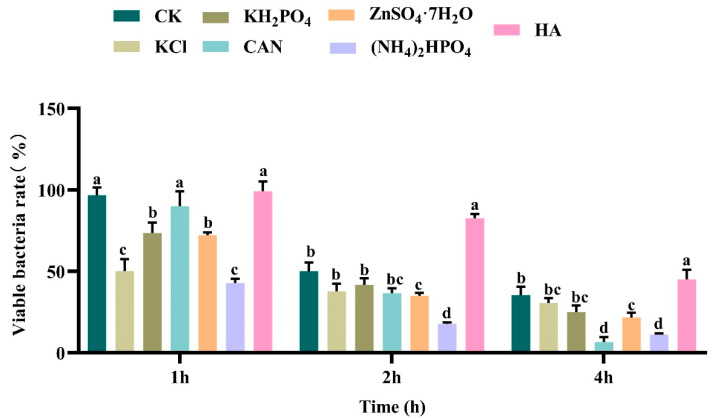
Effect of individual fertilizer on the UV resistance of 16,000 IU/mg Bt WP from Lv’an. The presence of the same letter indicated no significant difference, and the presence of different letters indicated a significant difference (*p* < 0.05).

Parallel experiments were also conducted with other commercial WPs (Figure A7, Figure A8 and Figure A9). Interestingly, different commercial WPs exhibited varying patterns of UV resistance enhancement in response to the fertilizer treatments. Notably, the WP from Kang’xin was particularly responsive to CAN treatment, while the WP from Lu’kang showed strong responses to KH_2_PO_4_ (see Appendix A for detailed results). These findings suggested that the commercial WPs interact differently with the fertilizers, leading to unique UV resistance enhancement profiles.

### 3.3. Fertilizers Boost the Insecticidal Activity of 32,000 IU/mg Bt WP from Lv’an

The fertilizers decreased the LC_50_ values of 32,000 IU/mg Bt WP from Lv’an from 0.434 g/L (CK without fertilizers) to 0.244 g/L, 0.264 g/L, 0.272 g/L, 0.328 g/L, and 0.336 g/L with the addition of KH_2_PO_4_, (NH_4_)_2_HPO_4_, ZnSO_4_·7H_2_O, HA, and KCl (Table 3). Therefore, the fertilizers can enhance insecticidal activity, except for CAN.

### 3.4. Determination of the Optimal Fertilizer Combination Using Response Surface Methodology and Evaluation of Its UV Resistance and Insecticidal Activity

*Optimized Fertilizer Combinations Enhanced the Biofilm Formation of Bt WPs via Response Surface Methodology*

To optimize the effects of fertilizers on the biofilm formation of Bt preparations, a systematic approach was employed involving sequential experimental designs. Firstly, the PB design was used to efficiently screen for the significant factors influencing the biofilm formation. The biofilm formation was quantified by measuring the optical density at 595 nm (OD_595_), which served as the response variable Y (Table 4). Regression analysis of the PB results (Table 5) yielded the following equation: Y = 1.88 + 0.1421A + 0.0794B − 0.1494C − 0.0289D + 0.0639E + 0.1516F. This linear model revealed the relative influence of each of the six fertilizers on biofilm formation by the 32,000 IU/mg Bt WP from Lv’an. The factors, in descending order of impact, were HA (factor F), ZnSO_4_·7H_2_O (factor D), KCl (factor A), KH_2_PO_4_ (factor B), (NH_4_)_2_HPO_4_ (factor E), and CAN (factor C). Notably, KCl and HA significantly promoted biofilm formation (*p* < 0.05), while CAN exhibited a negative effect. The reliability and significance of the PB model were evaluated. HA, ZnSO_4_·7H_2_O, and KCl were identified as key factors for further optimization of the concentrations using the steepest ascent method.

The Steepest Ascent Method allowed for efficient navigation of the response surface to pinpoint the region most likely to contain the optimal concentrations of fertilizers for maximizing biofilm formation. Coefficients from the PB analysis served as the step sizes, with signs indicating adjustment directions. This helped determine the optimal concentration range for enhanced biofilm formation, which was found to be between X + △ and X + 5△ (Table 6).

Based on the above results, KCl, ZnSO_4_·7H_2_O, and HA were selected as the key factors for further optimization using the BBD. The biofilm formation (measured as OD_595_) was used as the response variable (Y). The experimental results (Table 7) showed the specific combinations of the three fertilizers and their corresponding biofilm formation levels. Regression analysis of the data provided a quadratic model that described the relationship between the factors and the response, enabling the determination of the optimal concentrations for maximum biofilm formation.

The amount of BBF formation Y was used as the response value, and regression was fitted to the experimental data to establish regression simulation equations between the amount of BBF formation (Y) and the quantity of KCl, ZnSO_4_·7H_2_O, and HA: Y = 2.71 + 0.0367A + 0.0602B + 0.0488C + 0.0446AB + 0.0277AC + 0.0526BC − 0.1042A2 − 0.0854B2 − 0.1109C2. The results of regression simulation ANOVA are shown in Table 8. The quadratic regression model showed that the *p* value < 0.0001, indicating the model was significant. Its misfit term was 0.3813, showing that the model was well-fitted.

**Figure 3:**
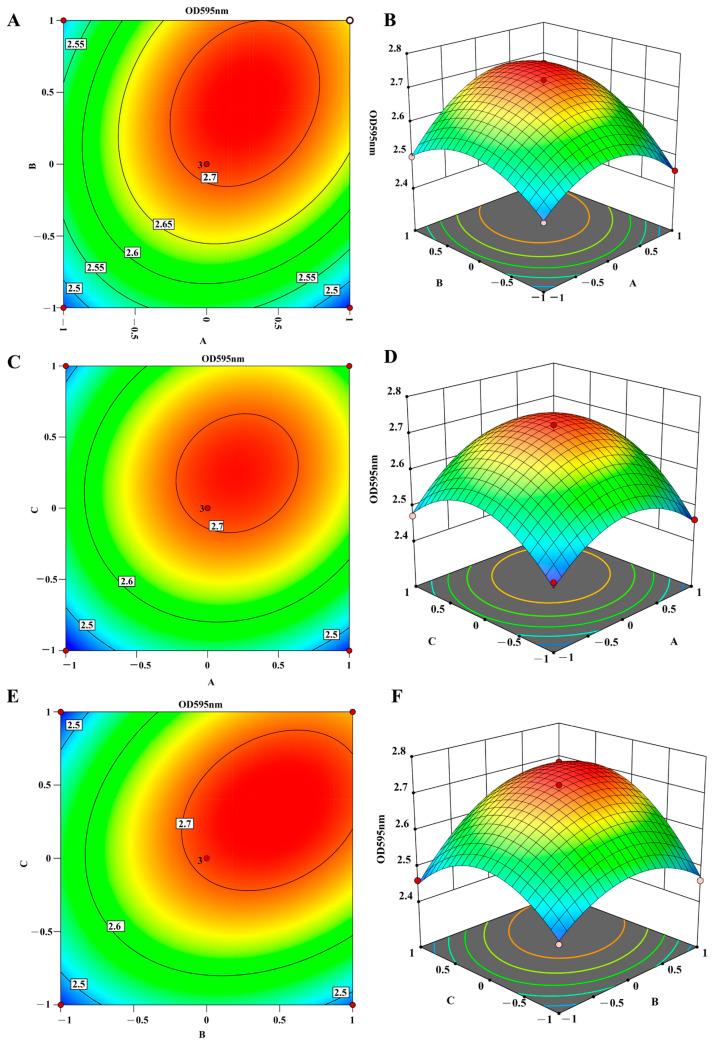
(**A**–**F**) Response surface and contour plots for optimization of fertilizer combinations on biofilm formation of Bt preparations. Colors range from blue to red, representing low to high OD values. A: KCl (g/L); B: ZnSO_4_·7H_2_O (g/L); C:. HA (μL/mL).

Based on the regression model ANOVA, three-dimensional (3D) surface and two-dimensional (2D) contour plots of the response surface quadratic model were generated using Design Expert software 13.0 (Figure 3). The shape of the surface plot revealed key interactions between the fertilizers. The elliptical shape indicated that the interaction between KCl (A) and HA (C) showed that HA (C) had a more pronounced effect on biofilm formation than KCl (A). Similarly, the interaction between HA (C) and ZnSO_4_·7H_2_O (B) revealed that HA (C) exerted a stronger influence on biofilm formation than ZnSO_4_·7H_2_O (B). In contrast, the interaction between KCl (A) and ZnSO_4_·7H_2_O (B) suggested that KCl (A) had a greater impact than ZnSO_4_·7H_2_O (B). The optimal combination of the three factors, which resulted in the maximum biofilm formation (OD_595_ value of 2.738), was achieved with 8.346 g/L of KCl, 2.751 g/L of ZnSO_4_·7H_2_O, and 25.681 μL/mL of HA.

*The compound fertilizer enhanced the UV resistance of 32,000 IU/mg Bt WP from Lv’an.*

**Figure 4:**
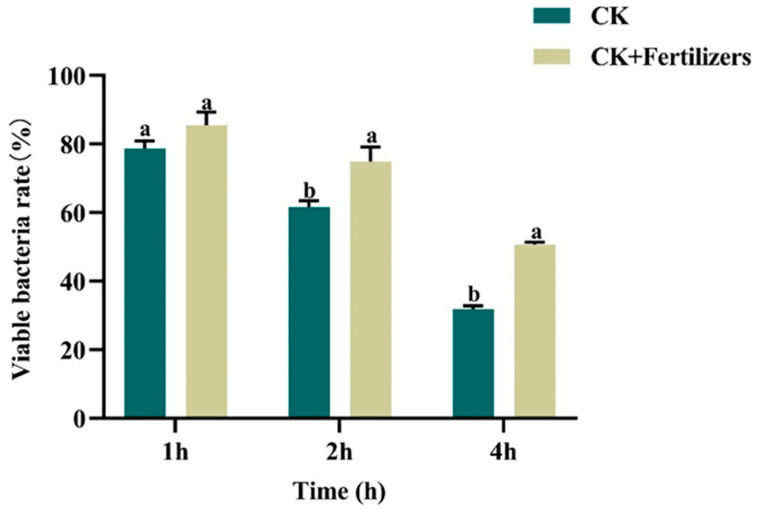
Effect of a compound fertilizer on the UV resistance of the 32,000 IU/mg wettable power. CK: Bt preparation without fertilizer addition. Fertilizers: a combination of KCl, ZnSO_4_·7H_2_O, and HA. The presence of the same letter indicated no significant difference, and the presence of different letters indicated a significant difference (*p* < 0.05).

The combination of KCl, ZnSO_4_·7H_2_O, and HA was found to significantly enhance the biofilm formation of the 32,000 IU/mg Bt WP from Lv’an (Figure 4). After 1 h of UV irradiation, there was a slight increase in UV resistance with this fertilizer combination. When the irradiation time was extended to 2 h, the experimental group showed a significant improvement in UV resistance compared to the control group (*p* < 0.05). After 4 h of UV irradiation, the fertilizer combination provided a marked improvement in UV resistance. These findings suggest that this specific fertilizer combination helps the 32,000 IU/mg Bt WP from Lv’an better withstand UV irradiation.

### 3.5. Compound Fertilizer Synergistically Enhances the Insecticidal Activity of 32,000 IU/mg Bt WP from Lv’an

The combination of 8.346 g/L of KCl, 2.751 g/L of ZnSO_4_·7H_2_O, and 25.681 μL/mL of HA showed the best promotion of the BBF formation for 32,000 IU/mg Bt WP from Lv’an. A compound fertilizer made with them was investigated for its effect on the insecticidal activity of the Bt WP (Table 9). The results with the addition of the compound fertilizer (the treatment) showed that the LC_50_ value was 0.243 g/L, while the LC_50_ value without the compound fertilizer (the CK) was 0.434 g/L. In the biofilm state, the LC_50_ values were 0.139 g/L and 0.291 g/L from the treatment and the CK, respectively. This indicated that the compound fertilizer could strongly help the Bt preparation to enhance the insecticidal activity of the Bt WP.

## 4. Discussion

This study shows that the enhancing effects of different fertilizers on Bt are dependent on the preparation type, with varying results across different Bt preparations. The optimized fertilizer combination significantly improved the biofilm formation, UV resistance, and insecticidal activity of Bt wettable powders. These findings highlight the potential of fertilizers as sustainable enhancers for microbial pesticides. However, further research is needed to better understand the interactions between fertilizers and Bt strains, as well as the strain-specific responses in different preparations, in order to optimize fertilizer application strategies.

These results suggest that fertilizer type and environmental conditions play crucial roles in determining the efficacy of Bt formulations. The varied responses of different Bt formulations to fertilizers are due to differences in their strain-specific metabolic pathways, nutrient requirements, and biofilm-forming abilities. Certain strains may better utilize nutrients like potassium or zinc, enhancing biofilm formation and UV resistance. These differences emphasize the need for tailored fertilizer applications for specific Bt formulations [22]. This study demonstrates that KCl, ZnSO_4_·7H_2_O, and HA significantly promote the biofilm formation of Bt preparations. Among these, the optimized fertilizer combination resulted in the highest OD_595_ value for biofilms. The data indicate that the promoting effects of different fertilizers are preparation-dependent, with HA showing a more universal effect across all preparations. Fertilizers enhance Bt biofilm formation through multiple mechanisms, including nutrient provision, regulation of osmotic pressure, and activation of signaling pathways. Potassium ions (K⁺) from KCl can regulate osmotic balance and activate membrane protein kinases, such as KinC, which promote extracellular polysaccharide (EPS) production and biofilm stability [27]. Humic acid (HA) provides a carbon source and enhances bacterial adhesion, while zinc ions (Zn²⁺) from ZnSO₄·7H₂O stabilize the biofilm matrix by binding to EPS and regulating metabolic pathways [28]. However, whether KCl directly activates the c-di-GMP signaling pathway to influence quorum sensing requires further validation [29]. HA, as a complex organic compound, was found to significantly promote biofilm formation by providing a carbon source, enhancing bacterial adhesion, and stabilizing the extracellular polysaccharide network [30,31]. The zinc ions in ZnSO_4_·7H_2_O regulate bacterial metabolic activity and protein synthesis capacity through the high-affinity zinc transport system (e.g., ZnuABC) and the zinc-responsive regulator Zur, thereby enhancing bacterial adaptability to adverse environments. Additionally, zinc ions may stabilize the biofilm matrix by binding to EPS and have been found to regulate signaling pathways associated with biofilm formation in certain bacteria, such as Pseudomonas aeruginosa [29,32]. These mechanisms may work together to further promote the formation and stability of Bt biofilms.

Beyond biofilm formation, the stability of molecular complexes, such as those involving γ-cadinene and COX-2, may also influence the overall performance of Bt preparations [33]. Even in the absence of hydrogen bonds, the stability of these complexes may be attributed to other interactions, including van der Waals forces, hydrophobic interactions, and π-π stacking [34]. These non-covalent interactions, though individually weaker, can collectively stabilize molecular complexes [35]. Further studies, such as molecular dynamics simulations, are needed to elucidate the precise mechanisms underlying this stability and its potential contribution to Bt efficacy [36].

Ultraviolet radiation is one of the main limiting factors affecting the field stability of Bt preparations [8]. This study further revealed that different types of fertilizers could significantly enhance the UV resistance of Bt preparations, although their effects varied depending on the preparation type and duration of UV exposure. The data showed that KCl exhibited a notable effect in enhancing UV tolerance in certain preparations, particularly under prolonged UV exposure, which might be related to the mechanism of potassium ions regulating bacterial osmotic pressure and protecting cell membrane structure [27]. Meanwhile, humic acid (HA) exhibited broad-spectrum UV protection across various Bt preparations, particularly in the Lv’an preparation. This protective effect may stem from HA enhancing the activity of antioxidant enzymes (such as superoxide dismutase and peroxidase), thereby reducing the accumulation of reactive oxygen species (ROS) and mitigating oxidative damage to bacterial cells caused by UV radiation [37]. In contrast, KH_2_PO_4_ exhibited a more pronounced UV protection effect in the Lu’kang preparation, which may be related to phosphates providing sufficient phosphorus for bacterial cells, thereby promoting nucleic acid repair and energy metabolism [38]. It is worth noting that the UV protection effects of different fertilizers exhibit time-dependent variations. These findings are consistent with previous studies, which indicated that nutrient supplementation could promote bacterial metabolism, thereby increasing the production of insecticidal crystal proteins (ICPs). The enhancement of Bt’s UV resistance by fertilizers likely involves multiple mechanisms. While HA and ZnSO₄·7H₂O significantly promoted biofilm formation (Figure 1), which can act as a physical barrier against UV radiation, other fertilizers like KH₂PO₄ and (NH₄)₂HPO₄ may improve UV resistance through alternative pathways, such as supporting nucleic acid repair and energy metabolism [39]. These findings suggest that the overall UV protection is a result of combined effects, including biofilm formation, nutrient provision, and cellular stabilization [40].

Finally, insecticidal activity is the core indicator of Bt preparation performance. The results showed that different fertilizers significantly enhanced the insecticidal efficacy of Bt preparations, with optimized compound fertilizers showing the most pronounced effect. However, this effect exhibited notable preparation dependency and fertilizer selectivity. These findings are consistent with previous studies, which indicated that nutrient supplementation can promote bacterial metabolism, thereby increasing the production of insecticidal crystal proteins (ICPs). Additionally, melanin precursors can stabilize Cry proteins and enhance their bioactivity under environmental stress [41]. Similarly, biofilms play a critical role in enhancing toxin persistence by stabilizing microbial communities in harsh environments, thereby maintaining toxin production and prolonging its effectiveness [42]. The observed synergistic effects of the compound fertilizers in this study represent a significant advancement.

The results further indicate that the synergistic effects of fertilizers are not limited to enhancing biofilm formation but also improve the UV resistance and insecticidal activity of Bt. This multifunctionality highlights the potential of fertilizers as sustainable enhancers for microbial pesticides. Moreover, compared to synthetic UV stabilizers, fertilizers provide a more sustainable approach, achieving comparable improvements in Bt durability and efficacy while reducing environmental impact [43]. However, the variability in the enhancement of insecticidal activity by different fertilizers across preparations suggests that their effectiveness depends not only on the chemical composition of the fertilizers but also on the strain characteristics and initial activity of the Bt preparations. This underscores the need to consider the synergistic or antagonistic interactions between fertilizers and preparations when optimizing fertilizer combinations.

While the new fertilizer preparations enhance the environmental persistence and efficacy of Bt preparations, potential trade-offs must be considered. Increased persistence may pose risks to non-target organisms, such as beneficial insects and soil microbiota, due to prolonged exposure to Bt toxins [44,45]. Additionally, the use of zinc and potassium-based fertilizers could lead to nutrient runoff or heavy metal accumulation, impacting soil and water quality [46,47]. The variability in Bt responses to fertilizers across different formulations highlights the importance of strain-specific optimization. To mitigate these risks, future studies should evaluate non-target effects, optimize application rates, and tailor fertilizer combinations to specific Bt strains. Balancing efficacy with environmental safety will be critical for the sustainable adoption of these preparations [45].

In conclusion, this study demonstrates that optimized combinations of KCl, ZnSO₄·7H₂O, and HA significantly enhance the biofilm formation, UV resistance, and insecticidal activity of Bt wettable powders. These findings highlight the potential of fertilizers as sustainable enhancers for microbial pesticides, offering a cost-effective and environmentally friendly alternative to synthetic UV stabilizers. Compared to complex encapsulation or expensive genetic modification methods, using fertilizers to enhance Bt performance is simpler and more cost-effective. Encapsulation physically shields Bt cells to improve UV resistance [48], while genetic modification enhances stress resistance through gene editing. Fertilizer methods, on the other hand, leverage natural nutrient interactions to promote biofilm formation, cellular stabilization, and metabolic resilience, reducing costs and aligning with sustainable agriculture. The findings of this study have significant practical implications for sustainable agriculture. Fertilizer-enhanced Bt formulations can be applied in integrated pest management (IPM) programs to control pests such as *Plutella xylostella* in cruciferous crops, where chemical pesticide resistance is widespread [49,50]. This approach not only enhances crop protection but also supports environmentally friendly farming practices. However, fertilizer formulations tailored to specific Bt strains and field conditions still require optimization research.

However, this study has limitations. The laboratory-based results require validation through field studies, and the interactions between Bt and the broader microbial community need further exploration [51]. Strain-specific responses also suggest the need for tailored fertilizer optimization based on metabolic and regulatory differences. Future research should also consider the stability of these formulations under diverse environmental conditions and assess their long-term impacts on ecosystems. Despite the promising results, several limitations must be addressed. Prolonged use of fertilizer-enhanced Bt formulations may lead to pest resistance, as observed with other microbial pesticides. Additionally, the potential non-target effects on beneficial insects and soil microbiota require further investigation [52,53]. Future research should focus on optimizing application rates and monitoring resistance development to ensure the long-term sustainability of this approach. 

Additionally, regulatory obstacles regarding the commercialization of fertilizer-enhanced Bt formulations in agriculture must be addressed, with a particular focus on safety standards and approval processes for natural fertilizers in pest management. More research into soil interactions and local environmental conditions will be essential for providing practical recommendations to agricultural practitioners. This would allow better decision-making regarding fertilizer selection and application, ensuring that Bt performance is maximized without negative ecological consequences.

In summary, this study lays the foundation for sustainable microbial pesticides by integrating natural fertilizers with microbial technology, addressing key challenges in agricultural pest management while promoting environmental sustainability.

## Figures and Tables

**Table 1:** The factors and the levels of PB design.

Code	Factors	Levels	
		−1	1
A	KCl	4 g/L	8 g/L
B	KH_2_PO_4_	1 g/L	1.25 g/L
C	CAN	2 g/L	6 g/L
D	ZnSO_4_·7H_2_O	1 g/L	4 g/L
E	(NH_4_)_2_HPO_4_	2 g/L	6 g/L
F	HA	10 μL/mL	25 μL/mL

**Table 2:** The factors and levels of BBD.

Variables	Factors	Levels		
		−1	0	1
A	KCl (g/L)	8.1	8.3	8.5
B	ZnSO_4_·7H_2_O (g/L)	2.5	2.7	2.9
C	HA (μL/mL)	25.2	25.6	26.0

**Table 3:** Effect of individual fertilizer on the insecticidal activity of 32,000 IU/mg Bt WP from Lv’an.

LA 320,000 IU/mg	LC_50_ (g/L)	95%CL	Slope	R^2^	Fold Increase
Bt	0.434	0.391–0.744	0.328	0.978	-
Bt + KCl	0.336	0.149–0.537	0.489	0.973	1.292
Bt + KH_2_PO_4_	0.244	0.011–0.514	0.216	0.855	1.479
Bt + CAN	0.437	0.232–0.690	0.384	0.938	-
Bt + ZnSO_4_·7H_2_O	0.272	0.015–0.334	0.617	0.971	1.596
Bt+ (NH_4_)_2_HPO_4_	0.264	0.012–0.299	0.684	0.951	1.644
Bt + HA	0.328	0.124–0.561	0.479	0.987	1.323

**Table 4:** Results of the PB experiment on the effects of six fertilizers on the biofilm formation of 32,000 IU/mg Bt WP from Lv’an.

Number	KCl (A)	KH_2_PO_4_ (B)	CAN (C)	ZnSO_4_·7H_2_O (D)	(NH_4_)_2_HPO_4_ (E)	HA (F)	OD_595_
1	−1	1	1	−1	−1	1	1.789
2	−1	−1	1	1	−1	−1	1.256
3	1	1	−1	1	−1	−1	2.011
4	1	−1	−1	−1	−1	1	2.200
5	−1	−1	−1	1	1	1	1.999
6	−1	1	−1	−1	1	−1	1.899
7	1	1	1	1	1	1	2.130
8	1	−1	1	−1	1	−1	1.739

**Table 5:** Regress variance results of the PB experiment on the effects of six fertilizers on the biofilm formation of 32,000 IU/mg Bt WP from Lv’an.

Source	Sum of Squares	df	Mean Square	F-Value	*p*-Value	
Model	0.6137	6	0.1023	291.32	0.0448	Significant
A-A	0.1616	1	0.1616	460.22	0.0297	
B-B	0.0504	1	0.0504	143.55	0.0530	
C-C	0.0067	1	0.0067	19.00	0.1436	
D-D	0.1785	1	0.1785	508.37	0.0282	
E-E	0.0326	1	0.0326	92.96	0.0658	
F-F	0.1839	1	0.1839	523.81	0.0278	
Residual	0.0004	1	0.0004			
Cor Total	0.6141	7				

**Table 6:** Results of the steepest ascent method experiment on the effects of three fertilizers on the biofilm formation of 32,000 IU/mg Bt WP from Lv’an.

Step Change Value	KCl (g/L)	ZnSO_4_·7H_2_O (g/L)	HA (μL/mL)	OD_595_
X	8	2	25	2.469
△	0.1	−0.1	0.2	-
X + △	8.1	2.9	25.2	2.112
X + 2△	8.2	2.8	254	2.299
X + 3△	8.3	2.7	25.6	2.369
X + 4△	8.4	2.6	25.8	2.308
X + 5△	8.5	2.5	26	2.276
X + 6△	8.6	2.4	26.2	2.162
X + 7△	8.7	2.3	26.4	2.002

**Table 7:** Results of the BBD experiment on the effects of three fertilizers on the biofilm formation of 32,000 IU/mg Bt WP from Lv’an.

Number	A	B	C	OD_595_
1	1	1	0	2.666
2	−1	0	1	2.472
3	0	1	−1	2.460
4	−1	−1	0	2.462
5	−1	0	−1	2.451
6	0	0	0	2.706
7	0	0	0	2.698
8	1	0	−1	2.461
9	0	−1	−1	2.448
10	1	0	1	2.593
11	−1	1	0	2.496
12	0	0	0	2.724
13	0	−1	1	2.461
14	0	1	1	2.684
15	1	−1	0	2.454

**Table 8:** Regression analysis of the BBD experiment on the effects of three fertilizers on the biofilm formation of 32,000 IU/mg Bt WP from Lv’an.

Source	Sum of Squares	df	Mean Square	F-Value	*p*-Value	
Model	0.1786	9	0.0198	73.53	<0.0001	Significant
A-A	0.0108	1	0.0108	40.04	0.0015	
B-B	0.0290	1	0.0290	107.41	0.0001	
C-C	0.0190	1	0.0190	70.47	0.0004	
AB	0.0079	1	0.0079	29.44	0.0029	
AC	0.0031	1	0.0031	11.36	0.0199	
BC	0.0111	1	0.0111	40.96	0.0014	
A2	0.0401	1	0.0401	148.46	<0.0001	
B2	0.0269	1	0.0269	99.82	0.0002	
C2	0.0454	1	0.0454	168.32	<0.0001	
Residual	0.0013	5	0.0003			
Lack of Fit	0.0010	3	0.0003	1.77	0.3813	Not significant
Pure Error	0.0004	2	0.0002			
Cor Total	0.1799	14				

**Table 9:** Effect of a compound fertilizer on the insecticidal activity of 32,000 IU/mg Bt WP from Lv’an.

Groups	LC_50_ (g/L)	95% CL	Slope	R^2^	Increased Fold
Bt	0.434	0.391–0.744	0.328	0.978	-
Bt WP added with a compound fertilizer	0.243	0.093–0.371	0.457	0.184	1.786
Bt WP in the BBF state	0.291	0.116–0.579	0.388	0.845	1.491
Bt WP added with a compound fertilizer in the BBF state	0.139	0.04–0.193	0.825	0.943	3.122

## Data Availability

The original contributions presented in this study are included in the article. Further inquiries can be directed to the corresponding authors.

## Abbreviations

The following abbreviations are used in this manuscript:

*Bacillus thuringiensis*	Bt
bacterial biofilms	BBF
wettable powders	WPs
Humic acid	HA
Zinc sulfate heptahydrate	ZnSO_4_·7H_2_O
Potassium chloride	KCl
Potassium dihydrogen phosphate	KH_2_PO_4_
Optical density	OD
Median lethal concentration	LC_50_

## Appendix A

### Appendix A.3. Effects of Fertilizers on the Insecticidal Activities of Bt WPs

**Table A1:** Effect of fertilizers on the insecticidal activity of 32,000 IU/mg Bt WP from Lu’kang.

Groups	LC_50_ (g/L)	95% CL	Slope	R^2^	Fold Increase
CK	0.582	0.301–0.780	0.133	0.918	-
Bt + KCl	0.745	0.323–0.915	0.088	0.976	-
Bt + ZnSO_4_·7H_2_O	0.564	0.368–0.838	0.323	0.924	1.032
Bt + KH_2_PO_4_	0.465	0.240–0.760	0.334	0.966	1.252
Bt + CAN	0.541	0.261–0.996	0.232	0.979	1.076
Bt+ (NH_4_)_2_HPO_4_	0.623	0.310–1.257	0.171	0.988	-
Bt + HA	0.372	0.114–0.297	0.633	0.968	1.565

**Table A2:** Effect of fertilizers on the insecticidal activity of 16,000 IU/mg Bt WP from Lv’an.

Groups	LC_50_ (g/L)	95% CL	Slope	Sig	Fold Increase
CK	0.534	0.360–0.520	0.526	0.954	-
Bt + KCl	0.237	0.038–0.238	1.047	0.960	2.253
Bt + ZnSO_4_·7H_2_O	0.445	0.186–0.562	0.492	0.753	1.200
Bt + KH_2_PO_4_	0.324	0.081–0.324	0.847	0.874	1.648
Bt + CAN	0.432	0.287–0.779	0.272	0.754	1.236
Bt+ (NH_4_)_2_HPO_4_	0.243	0.111–0.343	0.505	0.990	2.198
Bt + HA	0.412	0.217–0.759	0.289	0.951	1.296
